# Hypoglycemia and hyperglycemia in neonatal encephalopathy: A narrative review

**DOI:** 10.1097/MD.0000000000039488

**Published:** 2024-09-06

**Authors:** Sughra Asif, Maryam Shaukat, Kashaf Khalil, Hadiya Javed, Muhammad Safwan, Khadija Alam, Sabahat Fatima, Prishotam Chohan, Huraim Muhammad Hanif, Mohammed Mahmmoud Fadelallah Eljack, Muhammad Daim Bin Zafar, Muhammad Hasanain

**Affiliations:** a Saidu Medical College, Swat, Pakistan; b Aga Khan University, Karachi, Pakistan; c Jinnah Sindh Medical University, Karachi, Pakistan; d Dow Medical College, Karachi, Pakistan; e Liaquat National Hospital and Medical College, Karachi, Pakistan; f Karachi Medical and Dental College, Karachi, Pakistan; g Fazaia Ruth Pfao Medical College, Karachi, Pakistan; h Faculty of Medicine and Health Sciences, University of Bakht Alruda, Ad Duwaym, Sudan.

**Keywords:** Encephalopathy, Ischemic encephalopathy, Neonatal dysglycemia, Neonatal Hyperglycemia, Neonatal hypoglycemia

## Abstract

Neonatal encephalopathy (NE) is a serious condition with various neurological dysfunctions in newborns. Disruptions in glucose metabolism, including both hypoglycemia and hyperglycemia, are common in NE and can significantly impact outcomes. Hypoglycemia, defined as blood glucose below 45 mg/dL, is associated with increased mortality, neurodevelopmental disabilities, and brain lesions on MRI. Conversely, hyperglycemia, above 120 to 150 mg/dL, has also been linked to heightened mortality, hearing impairment, and multiorgan dysfunction. Both aberrant glucose states appear to worsen prognosis compared to normoglycemic infants. Therapeutic hypothermia is the standard of care for NE that provides neuroprotection by reducing metabolic demands and inflammation. Adjunct therapies like glucagon and continuous glucose monitoring show promise in managing dysglycemia and improving outcomes. Glucagon can enhance cerebral blood flow and glucose supply, while continuous glucose monitoring enables real-time monitoring and personalized interventions. Maintaining balanced blood sugar levels is critical in managing NE. Early detection and intervention of dysglycemia are crucial to improve outcomes in neonates with encephalopathy. Further research is needed to optimize glycemic management strategies and explore the potential benefits of interventions like glucagon therapy.

## 1. Introduction

Neonatal encephalopathy (NE) refers to a range of neurological dysfunctions that occur in newborn infants within the first few days of life. They include altered mental status, abnormal muscle tone, seizures, respiratory and feeding difficulties. NE is influenced by various factors like preconception risks, maternal antepartum and intrapartum conditions, placental issues, hypoxia-ischemia (HI), perinatal infections, neonatal stroke or thrombophilia, metabolic disorders, and genetic or epigenetic abnormalities, prolonged rupture of membranes, and intrauterine growth restriction (IUGR).^[1]^ Other factors that contribute to the occurrence and severity of this disease include birth asphyxia, maternal infections, genetic predispositions, maternal health issues, and birth injuries. It is estimated that 2 to 6 per 1000 term births are impacted by neonatal encephalopathy, with hypoxic-ischemic encephalopathy (HIE) representing 1.5 per 1000 term births.^[2]^

The terms NE and HIE are used to refer to a full-term infant with abnormal neurological findings at birth and indications of perinatal oxygen deprivation or ischemia. However, HIE is actually a specific subset of NE.^[3,4]^ The ACOG-AAP classification of NE highlights neonatal symptoms and related elements that suggest acute peripartum or intrapartum HI as probable causes of acute encephalopathy. These neonatal indicators include an Apgar score of <5 at both 5 and 10 minutes, fetal umbilical acidemia (with a pH <7.0 or a base deficit of 12 mmol/L or more), MRI or magnetic resonance spectroscopy neuroimaging findings of acute brain injury that align with hypoxic-ischemic conditions, and evidence of dysfunction in multiple organs. Other contributing elements include a notable hypoxic or ischemic incident occurring immediately before or during labor and delivery, fetal heart rate that points to an acute intrapartum/peripartum issue, findings of brain injury that correspond with an acute redelivery or delivery-associated cause, and conditions affecting development, such as spastic quadriplegia and cerebral palsy.^[5]^ In contrast to HIE, NE has diverse causes and is associated with a broad spectrum of contributing factors.^[6]^ NE has been linked to factors such as abnormal placental development, a history of seizures in the family, treatments for infertility, maternal thyroid disorders, low income lifestyle, and congenital defects.^[7–9]^ Other predisposing factors validated in the literature include growth abnormalities of the fetus, unusual head circumference, significant infarction of the placenta, and substantial infections.^[10,11]^

While there may be variations for specific guidelines for neonatal encephalopathy, comprehensive guidelines have been established for the management of HIE. Organizations such as the Canadian Paediatric Society and the Queensland Maternity and Neonatal Clinical Guidelines provide evidence-based recommendations for the diagnosis, assessment, and management of HIE. These guidelines focus on various aspects of care, including therapeutic hypothermia, seizure monitoring, supportive care, and long-term follow-up.^[12]^ The intervention for neonatal encephalopathy involves inducing mild hypothermia within 6 hours of birth for near-term and full-term infants to minimize brain damage and improve outcomes.^[13]^

Glucose is the main source of energy for the developing brain, and disruptions in its metabolism leads to damage to brain cells. After birth, the supply of glucose through the placenta stops, and maintaining normal levels of glucose in the body relies on the liver and glycogen stores, hormonal control, and the intake of nutrients either orally or through intravenous means.^[14]^ Hypoglycemia refers to blood glucose levels dropping below 45 mg/dL in newborns. Key risk factors for neonatal hypoglycemia include being born preterm, high birth weight, or low birth weight, and being an infant of a diabetic mother^[15]^ (Table 1). Severe neonatal hypoglycemia leads to neurologic damage, mental retardation, epilepsy, impaired cardiac performance, and muscle weakness (Table 1). Hyperglycemia, on the other hand, refers to elevated blood glucose levels, above 120 to 150 mg/dL in newborns. Various thresholds of blood glucose levels (7, 8.3, 10, and 12 mmol/L) have been used to define neonatal hyperglycemia. In neonates has been associated with a heightened likelihood of death or neuro-disability. Both hypoglycemia and hyperglycemia are modifiable risk factors since they are prevalent in NE.^[6]^ This literature review aims to study the relationship between dysglycemia in neonatal encephalopathy, review the clinical outcomes observed in neonates with hypoglycemia and hyperglycemia, and studies the available therapeutic options.

**Table 1:** Risk factors for hyperglycemia and hypoglycemia in neonates.

Hyperglycemia	Hypoglycemia
Mother exceeding 120% of the ideal body weight	Late preterm infants
Family history of type 2 diabetes	Large-for-gestational age
Maternal hyperlipidemia	Small-for-gestational age
Maternal hypertension	Placental insufficiency-intrauterine growth restriction (PI-IUGR)
History of gestational diabetes	Infant of a diabetic mother (IDM)
Maternal polycystic ovarian syndrome (PCOS)	

## 2. Methods

A comprehensive search of the following electronic databases was performed: PubMed Central and ClinicalTrials.gov. Search terms used were Neonatal Encephalopathy OR Hypoxic Ischemic Encephalopathy, which were combined with hypoglycemia, hyperglycemia, dysglycemia. Duplicates were excluded prior to retrieval of references. Abstracts for each reference were obtained and screened. Studies were eligible for inclusion if they were written in English and were human-based. Studies were first selected, looking for a presence of a clinical association between “neonatal encephalopathy,” “hypoxic-ischemic encephalopathy,” and “dysglycemia,” “hyperglycemia,” “hypoglycemia,” and reporting the details of glycemic management interventions. No publication date limits were set. The title and abstracts of studies were independently examined by 2 authors (K.K. and H.J.) and were critically checked by 2 independent reviewers (M.H. and M.D.); conflicting viewpoints were discussed until a consensus was reached.

### 2.1. Perinatal glucose metabolism and utilization of other substrates

Glucose serves as the primary source of energy for the fetal and newborn brain. Research in both animals and humans suggests that initially, cerebral glucose utilization is low but increases as the brain matures. The rise in glucose utilization corresponds to increased brain activity and energy requirements. In instances of hypoglycemia, alternative energy sources like ketone bodies and lactate substitute for glucose and provide protection to the developing brain.^[16]^

A set of mechanisms regulates glucose delivery to these tissues to maintain its metabolism. During the latter half of gestation, the fetus experiences substantial growth with a need for an increased transfer of glucose from the placenta to satisfy the growing metabolic demands. The augmentation in placental glucose transport happens through 2 mechanisms: (i) as the fetal glucose concentration decreases in comparison to maternal glucose concentration, the concentration gradient for glucose across the placenta rises,^[17]^ (ii) the capability of placental transport increases.^[18]^

The fetus’s developmental conditions rely on maternal nutritional and metabolic status and the functioning of the placenta. The majority of nutrients need to be transferred from the maternal blood circulation through the placenta to the fetus. Additionally, the placenta releases hormones and growth factors that significantly influence maternal metabolism. Glucose transporters (GLUTs) facilitate glucose transport across the placenta, moving glucose from maternal to fetal circulation. They are found in both the maternal-facing microvillous plasma membrane (MVM) and the fetal-facing basal plasma membrane of the syncytiotrophoblast. Various isoforms of GLUTs, including GLUT1, GLUT3, GLUT4, GLUT8, GLUT9, GLUT10, and GLUT12, are expressed in the human placenta.^[19]^ GLUT4 is regulated by insulin that is primarily found in conventional insulin-responsive tissues like adipose tissue, skeletal muscle, and cardiac muscle. While it has been noted that GLUT4 expression occurs in intravillous stromal cells of the human placenta,^[20]^ earlier research indicated that GLUT4 was not detected in the syncytiotrophoblast at full term.^[21]^ On the contrary, GLUT4 protein expression is reported in both the cytoplasm and MVM of syncytiotrophoblast during the first trimester. Desoye et al^[22]^ utilized immunohistochemistry to show that insulin receptors in the placenta were mainly present in the MVM of the syncytiotrophoblast during early pregnancy. However, by term, insulin receptor protein expression was predominantly observed in the vessels of the fetoplacental circulation. Ericsson et al^[23]^ found that insulin enhances glucose uptake in placental villous explants during the first trimester, indicating that insulin can regulate glucose transport in the placenta during early pregnancy. The fetus does not produce its own glucose as maternal glucose supply and transfer across the placenta adequately meet the fetal glucose needs.

In fetuses with placental insufficiency-intrauterine growth restriction, there is a continual and gradual decline in both glucose and oxygen delivery, along with lowered plasma insulin levels and increased plasma concentrations of norepinephrine, cortisol, and glucagon.^[24]^ Prolonged hypoxemia lasting for 1 to 2 weeks results in high norepinephrine levels in the fetus, which signals the activation of hepatic glucose production. Extended exposure to anemic hypoxemia for a period of 9 or more days leads to elevated levels of cortisol and glucagon in the fetus, upregulates the expression of hepatic PCK1 and reduces the glycogen content in the fetal liver.^[25]^ In the fetuses of mothers with obesity, insulin resistance in the peripheral tissues begins to develop during the prenatal period.^[26]^ Chronic hyperinsulinemia observed in insulin-resistant mothers, along with corresponding high fetal insulin levels induce insulin resistance in the fetal brain.^[27]^ In contrast to infants born to nondiabetic mothers, children born to diabetic mothers with poorly managed blood sugar levels exhibit neurophysiological deficits and are at increased risk of developing metabolic syndrome, obesity, and type 2 diabetes later in life.^[28]^ A study indicates that prenatal exposure to alcohol leads to increased expression of genes involved in glucose production in the liver of the fetus. It causes a tendency for increased levels of glucose in the blood and liver, while glucose levels in the fetal brain and placenta remain low. Consequently, there is a decrease in maternal fasting glucose, a reduction in liver glucose production, and an increase in the rate at which glucose is cleared from the bloodstream. These metabolic alterations are associated with reduced fetal body and brain weight.^[29]^

After birth, when the umbilical cord is clamped, the constant supply of glucose and nutrients from the placenta abruptly stops, and the newborn’s own internal glucose metabolism starts. During the first few hours of life, plasma glucose levels go through a transitional phase before reaching a normal physiological low point. This stimulates the production of glucose within the healthy, full-term newborn.^[30]^ On the other hand, premature or small-for-gestational-age (SGA) infants have an immature and underdeveloped hormonal and metabolic adaptation process. The physiologic decrease in plasma glucose levels activates the glycogen phosphorylase enzyme, which promotes the release of glucose from the liver through glycogenolysis and helps the regulation of glucose levels in the initial hours after birth.^[31]^ Epinephrine facilitates further glucose release from the liver. However, during prolonged periods of low plasma glucose, growth hormone and cortisol are also released. These hormones help mobilize fat and protein substrates and provide alternative energy sources for cerebral function.^[32]^

During prolonged hypoglycemia, neonatal hepatic glucose production is inadequate to meet the energy needs of the neonatal brain. As a primary compensatory mechanism, cerebral blood flow rises; however, despite this increase, neuronal cells cannot extract enough glucose from the low plasma glucose concentrations to meet their metabolic requirements.^[33]^ When physiological disruptions result in insufficient defenses against prolonged hypoglycemia, the consequences for the brain are severe and may cause permanent brain injury in neonates. Neonatal hypoglycemic brain injury arises when local energy reserves and alternative substrate supplies are exhausted. There is a disruption of neurotransmitter synaptic functions and dysregulation in the release of excitotoxins, which causes cytotoxic edema and neuronal cell death in specific brain regions such as the parietal-occipital and thalamic areas.^[34]^

### 2.2. Prevalence of dysglycemia in newborns with neonatal encephalopathy

“Dysglycemia” is a term utilized to characterize the variations in plasma glucose levels, encompassing both elevated (hyperglycemia) and diminished (hypoglycemia) levels. Additionally, it may denote conditions such as impaired glucose tolerance or impaired fasting glucose.^[35]^

Normal fasting blood glucose concentration typically falls within the range of 70 mg/dL (3.9 mmol/L) to 100 mg/dL (5.6 mmol/L). According to the American Diabetes Association, hypoglycemia occurs when blood glucose levels drop below 70 mg/dL.^[36]^ Hyperglycemia is defined as having blood glucose levels exceeding 125 mg/dL during fasting and surpassing 180 mg/dL 2 hours after a meal.

Hypoglycemia can be categorized into primary and secondary forms. Primary hypoglycemia refers to cases where it is the main reason for hospital admission, while secondary hypoglycemia denotes instances where it occurs during the hospital stay, known as inpatient hypoglycemia. Inpatient hypoglycemia can stem from diverse risk factors, including personal susceptibilities as well as institutional factors. These may encompass advanced age, concurrent health conditions, diabetes type, prior hypoglycemic episodes, body mass index, intensive glucose-lowering treatments, insufficient glucose monitoring, unclear medical directives, staffing and facility limitations, extended fasting, and mismatches between nutritional intake and treatment protocols.^[37]^

Following severe traumatic brain injury, there is a high occurrence of hyperglycemia, which is linked to unfavorable clinical outcomes and heightened mortality rates.^[38]^ Perioperative hyperglycemia is frequently observed in patients undergoing joint replacement surgeries.^[39]^ In individuals with genetic predispositions and lifestyle-related risk factors, aging contributes to the onset of type 2 diabetes by causing dysfunction in β-cells and hampering their ability to adapt to insulin resistance.^[40]^ Other major risk factors for hyperglycemia include: 1. exceeding 120% of the ideal body weight, 2. family background of type 2 diabetes, 3. individuals of Native American, Hispanic, Asian American, Pacific Islander, or African American descent, 4. having hyperlipidemia or hypertension, 5. previous experience of gestational diabetes, and 6. being diagnosed with polycystic ovarian syndrome.

A population-based cohort study examined 545 patients with moderate-to-severe NE. Among them, 199 newborns (36.5%) had normal blood sugar levels, 74 (13.6%) were hypoglycemic, 213 (39.1%) were hyperglycemic, and 59 (10.8%) showed glycemic instability, based on 2593 blood sugar measurements. The primary adverse outcome was observed in 45.8% of normoglycemic newborns, 59.7% of those with hypoglycemia, 67.5% of those with hyperglycemia, and 70.2% of those with glycemic instability (*P* < .01). The study found that dysglycemia impacts nearly two-thirds of infants with NE and is independently linked to an increased risk of mortality and/or brain lesions detected on magnetic resonance imaging.^[41]^ Another study conducted on neonates with HIE who underwent both therapeutic hypothermia and continuous glucose monitoring (CGM) found that dysglycemia was most commonly observed within the first 24 hours of therapeutic hypothermia, with hyperglycemia being more frequent. Continuous glucose monitoring revealed significant differences in the duration, frequency of episodes, and area under the curve of hypoglycemia among neonates with varying outcomes. Throughout the therapeutic hypothermia process, both hypoglycemia and early glycemic instability were strongly linked to unfavorable outcomes.^[42]^ A study involving 2000 newborns, who were born between 23 and 42 weeks of gestation, found that 19% experienced low levels of glucose in the blood (<45 mg/dL) within the first 3 hours of life, 6% of which experiencing severe hypoglycemia (<35 mg/dL).^[43]^ Another study employing both CGM and occasional glucose tests in the plasma revealed that 39% of normal full-term newborns experience an instance of low blood sugar (defined as < 47 mg/dL) within the first 4 days of life.^[44]^ In a study of 514 infants including preterm, infants of diabetic mothers, and those small or large for their gestational age 37.4 (1.7) weeks, standardized glucose screening revealed a 51% rate of hypoglycemia (defined as blood glucose < 47 mg/dL) in the first 48 hours following birth. 19% of high-risk infants experienced severe hypoglycemia (blood glucose < 36 mg/dL), and the incidence rate was consistent among all risk groups. The majority of hypoglycemia episodes (81%) happened on the first day of life, with 37% occurring after 3 normal blood glucose readings. Six percent had their first episode of hypoglycemia on day 2, which falls outside the typical hypoglycemia screening period in neonatal nurseries and neonatal intensive care units (NICUs). On the other hand, severe hypoglycemia usually occurred soon after birth, with 74% of cases happening within the first 6 hours. Nineteen percent of newborns experienced recurrent hypoglycemia, with the majority (70%) within their first 24 hours.^[45]^ A high fetal exposure to glucose due to maternal hyperglycemia is increasingly acknowledged as a potential factor contributing to adverse neurodevelopmental fetal outcomes, which may manifest in the later stages of life.^[46]^

Roughly 17% of pregnancies worldwide are impacted by hyperglycemia. After premature delivery, over 50% of infants with very low birth weight and up to 80% of infants with extremely low birth weight experience hyperglycemia within the initial 2 weeks.^[47]^ Between 43% and 54% of full-term newborns treated for HIE experience hyperglycemia.^[48]^

Many studies have confirmed the link between neonatal hypoglycemia and the risk of brain injury. Substantial evidence supports the occurrence of brain injury in newborns with genetic hyperinsulinism who experience hypoglycemia.^[49]^ Simultaneous hypoglycemia exacerbates brain injury resulting from HIE. Severe hypoglycemia (blood glucose levels < 36 mg/dL) and recurrent episodes (3 or more) are linked to a higher risk of impairments in visuo-motor and executive functions.^[50]^ The impact of hyperglycemia on neurological development directly relies on factors such as when it occurs, how severe it is, and how long it lasts. Early onset hyperglycemia in preterm newborns is linked to short-term neurological consequences, including hemorrhage within the ventricles and imaging showing reduced white matter.^[51]^

In a retrospective trial, neonates with HIE whose mothers had diabetes exposure showed a significantly lower gestational age at birth (38.6 weeks vs 39.7 weeks, *P* = .005) and a notably higher average birth weight (3588 ± 752 g vs 3214 ± 514 g, *P* = .012). Infants born to diabetic mothers with HIE required prolonged ventilation (8 days vs 4 days, *P* = .0047) and had extended stays in the NICU (18 days vs 11 days, *P* = .0483). They took a longer duration to achieve full oral feeding (15 days vs 7 days, *P* = .0432) compared to neonates born to nondiabetic mothers.^[52]^ Offspring of mothers with gestational diabetes face increased chances of being born with macrosomia and experiencing neonatal hypoglycemia.^[53]^ Additionally, preterm infants face a heightened risk of hyperglycemia due to a combination of environmental factors such as exposure to drugs, parenteral glucose infusion, sepsis, and IUGR, as well as intrinsic factors like hormonal regulation alterations leading to decreased insulin production and reduced suppression of hepatic glucose production.^[54,55]^

#### 2.2.1. Hypoglycemia and neonatal encephalopathy

While in periods of starvation, ketone bodies may provide an alternative temporary source of energy for the brain, long-term glucose deprivation leads to detrimental effects depending on the duration of the hypoglycemic state. Mild to moderate hypoglycemia may produce synaptic disturbances, whereas severe hypoglycemia may lead to neuronal death and cognitive impairment.^[56]^ Neonatal hypoglycemia under normal body conditions adversely affects neurodevelopment. Information processing, ion gradient maintenance across neuronal membranes, neural computation and glutamate vesicle production account for the majority of brain energy utilization. Lack of glutamate vesicles further decreases ATP levels, which inhibits all glutamate-dependent neuronal activity.^[57]^

As reported by a study, recurring neonatal hypoglycemia was substantially linked with ongoing neurodevelopmental and physical growth impairments until the age of 5.^[58]^ Alternatively, in a prospective cohort study conducted at a pediatric academic referral hospital, neonates born at full term with encephalopathy were enrolled within 6 hours of birth. The study included 45 infants (with a mean gestational age of 39.5 ± 1.4 weeks) of which 24 were male. During continuous aEEG monitoring, 16 episodes of hypoglycemia were observed in 9 infants, with a median duration of 77.5 minutes (maximum 220 minutes). However, hypoglycemic episodes did not show any association with aEEG changes.^[59]^ In the context of brain trauma, hypoglycemia compromises energy metabolism, exacerbates neuronal damage, further impairs cognitive function, and hinders recovery processes. Further, hypoglycemia within 24 hours after birth has been associated with motor and cognitive impairment at 1-year follow-up.^[60]^ Severe or prolonged hypoglycemia during the neonatal period can also lead to long-term impairments, including deficits in attention, memory, executive function, and overall cognitive development.^[57]^ These deficits may persist into childhood and adulthood, impacting academic achievement, social interactions, and quality of life. Neurons in the developing brain are actively proliferating, migrating, and forming synaptic connections. Hypoglycemia can compromise the energy supply to these developing neurons, leading to impaired synaptic plasticity and connectivity, which are crucial for normal cognitive development. Hypoglycemia in neonates may also result in delayed myelination in deep and periventricular white matter (particularly in the parieto-occipital lobe) and even cortical atrophy (particularly in the occipital lobe).^[61,62]^

Glutamate is the primary neurotransmitter in the brain. Hypoglycemia-induced extreme depolarization leads to increased extracellular glutamate levels in the synaptic space, activating a multitude of enzymes, including protein kinase C, Ca2+/calmodulin-dependent protein kinase II, phospholipases, proteases, phosphatases, nitric oxide synthase, endonucleases, etc.^[63]^ These enzymes further induce other excitotoxic cascades, leading to more glutamate release and eventually the generation of a positive feedback loop.^[54]^ This leads to metabolic acidosis, mitochondrial injury, free radical generation, and the auto-digestion of enzymatic breakdown products.^[64]^ Mitochondria from a brain experiencing hypoglycemia shows an enhanced ability to produce ROS when exposed to glutamate-induced excitotoxicity.^[57]^ Mitochondrial dysfunction, along with the production of ROS, increases oxidative stress within the neurons, which eventually accumulates into neuronal damage and progression to brain injury.^[64]^

Neonatal hypoglycemia is a major factor in neonatal mortality.^[65]^ It is also a major element in neonatal encephalopathy. The epidemiology and risk factors for neonatal hypoglycemia in neonatal encephalopathy involve various aspects. Vulnerable newborns, such as those with large-for-gestational age, SGA, born to diabetic mothers, or late preterm infants, are particularly at risk. Specifically, hypoglycemia rates are approximately 47% in large-for-gestational age infants, 52% in SGA infants, 48% in neonates of diabetic mothers, and 54% in late preterm infants.^[64]^ Moreover, for infants born before 33 weeks, the prevalence of hypoglycemia is nearly 34%.^[57]^

#### 2.2.2. Hyperglycemia and neonatal encephalopathy

The causes of hyperglycemia in premature infants include dextrose infusion for improved nutrition, diminished sensitivity to insulin, impaired function of pancreatic islet β-cells, and irregular levels of counter-regulatory hormones.^[66]^ Common complications in the NICU, such as sepsis, can further induce or worsen hyperglycemia in very low birth weight infants.^[67]^ Hyperglycemia modifies brain architecture and correlates with poorer neurodevelopmental results. In a prospective cohort study conducted at a pediatric academic referral hospital, neonates born at full term with encephalopathy were enrolled within 6 hours of birth. The study included 45 infants (with a mean gestational age of 39.5 ± 1.4 weeks). During continuous aEEG monitoring, 18 episodes of hyperglycemia were detected in 13 infants, with a median duration of 237.5 minutes (maximum 3125 minutes).Epochs of hyperglycemia were linked with poorer aEEG background scores (B 1.120, 95% CI 0.501–1.738, *P* < .001), reduced sleep–wake cycling (B 0.587, 95% CI 0.417–0.757, *P* < .001), and increased incidence of electrographic seizures (B 0.433, 95% CI 0.185–0.681, *P* = .001), even after adjusting for the severity of HI. The study concluded that in neonates with encephalopathy, periods of hyperglycemia were temporally associated with poorer overall brain function and increased occurrence of seizures, independently of HI severity.^[59]^

High glucose levels cause the blood–brain barrier to become more permeable, enabling glucose and other harmful elements to penetrate the central nervous system.^[68]^ Glucose interacts with compounds inside and outside the cell leading to the production of free radicals. This process results in oxidative stress, leading to alterations in cellular molecules, thereby compromising their functionality. Mitochondrial dysfunction and cellular damage ensue as a consequence of these modifications.^[69]^

If it happens at an early stage, the resultant changes in brain structure could render it more vulnerable to subsequent events in long-term conditions like Diabetes Mellitus Type 1. Long-term hyperglycemia can cause advanced glycation end products to form and potentially lead to neurodegenerative damage.^[70]^ Furthermore, elevated glucose levels can alter the makeup of sphingolipids resulting in reorganizations of cell membrane.^[71]^ Short-term complications of perinatal hyperglycemia include alterations in white matter imaging and occurrences of hemorrhages within ventricles.^[14]^ A study on full-term newborns with moderate-to-severe HIE who received therapeutic hypothermia found that those with hyperglycemia were more prone to damage in the basal ganglia or widespread injury patterns than those with normal glucose levels in the blood.^[72]^

In premature children, early exposure to elevated glucose levels correlates with imaging showing reduced white matter, increased susceptibility to developing intraventricular hemorrhage, and smaller head circumference.^[73]^

In full-term infants, early hyperglycemia in the first few hours after birth is linked to an increased risk of moderate/severe cerebral palsy, poor gross motor skills, and the development of seizures.^[59]^

Because of various environmental factors such as drug exposure, glucose administered parenterally, widespread infection, and IUGR, along with internal issues like hormonal dysregulation leading to decreased production of insulin and limited control over liver glucose production, preterm newborn face a higher likelihood of developing hyperglycemia.^[54]^ Neonatal diabetes mellitus (NDM) is an infrequent cause of elevated blood glucose levels in neonates. Unlike type 1 diabetes resulting from a combination of genetic predisposition and environmental factors, NDM results from monogenic defects that affect cellular channels like the K_ATP_ channel, ABCC8, or involve alterations in the INS gene. NDM affects about 1 in every 90,000 to 160,000 alive newborns^[74]^ and must be taken into account among infants who display insulin-dependent high blood glucose levels persisting for longer than 7 to 10 days without an alternative explanation.^[75]^

### 2.3. Observations

Several outcomes have been associated with variations in glycemic profile amongst patients suffering from neonatal encephalopathy. These include acid-base balance, liver function, renal function, hematological parameters, cardiorespiratory function, neurodevelopmental disability, and death. Herein, we summarize the findings concluded for the more widely reported parameters. An overview of the outcomes reported by the clinical trials referred to in this study is described in Table 2.

**Table 2:** The outcomes reported by the clinical trials.

Study author	Year	Gestational age, mean (SD)				Outcomes
		Hypoglycemic	Normoglycemic	Hyperglycemic	Glycemic liability	
Basu K Sudeepta et al	2016	38.8 (±1.8)	39.0 (±1.5)	39.3 (±1.5)	–	● Highest mortality rates in hypoglycemic infants, followed by normoglycemic, and hyperglycemic ● Significantly lower pH and bicarbonate levels, higher AST and ALT levels, higher prothrombin time, higher creatinine, and lower urine output during the first 24 hours in hypoglycemic group
Guellec Isabelle et al^[41]^	2023	39.1 (±1.7)	39.0 (±1.6)	38.9 (±1.6)	38.9 (±1.5)	● Highest mortality rates in glycemic liability group, followed by hyperglycemic, hypoglycemic and then normoglycemic neonates ● Highest aOR for brain lesions in hypoglycemic group, followed by hyperglycemic, glycemic liability and eventually normoglycemic. ● Lesions predominately observed in white matter, cortex, basal ganglia and thalami, posterior limb internal capsule
Tam EWY^[60]^^,^*	2012	39.8 (±1.4)	39.6 (±2.3)			● Increased OR of corticospinal tract injury in hypoglycemic neonates ● Increased OR of one-point worsened neuromotor score ● Hypoglycemia associated with 15 point lower cognitive and language score on the Bayley Scales of Infant Development
Lee IC et al^[76]^^,^†	2021	38.3 (±2.0)	38.7 (±1.3)	38.6 (±1.2)	-	● High incidence of basal ganglia, thalamus, and brain stem lesions observed in hyperglycemic followed by hypoglycemic neonates. ● High incidence of neurodevelopmental disability observed in hyperglycemic followed by hypoglycemic and normoglycemic neonates.
Parmentier CEJ et al^[77]^^,^*	2022	40.0 (1.86)	40.0 (2.79)			● Higher brain injury scores, lower 2-year motor scores, and lower motor and cognitive scores at preschool age observed in hypoglycemic neonates.
Filan PM et al^[78]^^,^‡	2006	38	–	–	–	● Parietal and occipital lobe brain lesions in hypoglycemic group
Barkovich AJ et al^[33]^^,^‡	1998	–				● Parietal and occipital lobe brain lesions in hypoglycemic group
Nadeem M et al^[48]^^,^§	2011	39 (1.5)				● Early hypoglycemia (0 to 6 hours of life) associated with abnormal neurodevelopmental outcome on 24 months of age. Association not significant when adjusted for HIE grade.

* Study categorized neonates in hypoglycemia and no hypoglycemia groups.

† Study categorized neonates into 3 groups based on blood glucose levels: >0 mg/dL and <60 mg/dL, ≥60 mg/dL and <150 mg/dL, ≥150 mg/dL. Our table considers these as hypoglycemic, normoglycemic, and hyperglycemic, respectively, to main uniformity.

‡ Study was a case series focusing on 4/5 cases of hypoglycemic neonates only.

§ Study only quoted overall gestational age for cohort.

#### 2.3.1. Mortality

A consistent theme across studies^[13,41,79]^ is the correlation between aberrant glucose levels, whether elevated or reduced, and heightened mortality rates. In a study by Guellec Isabelle et al, neonatal mortality was 40.7%, the highest, for the glycemic liability group (defined as at least 1 episode of hypoglycemia and 1 of hyperglycemia), 31.5% for the hyperglycemic group, 16.2% for the hypoglycemic group and 9.1% for the normoglycemic group.^[41]^ A study by Basu K Sudeepta et al, concluded that the rate of death and/or severe neurological disability at 18 months was 83% for hypoglycemic and 68% for hyperglycemic infants, compared with 49% in normoglycemic infants.^[13]^ A post hoc analysis of the high-dose erythropoietin for asphyxia and encephalopathy trial suggested increased aOR for death and neurodevelopmental impairment in both hypoglycemic and hyperglycemic neonates (2.62; 1.47–4.67 and 1.77; 1.03–3.03) in comparison to those with normal glucose levels.^[79]^ Thus, it may be concluded that it is the deviation of glucose levels from the norm which adversely impacts the continuation of life, more importantly than the direction of the fluctuation.

#### 2.3.2. Neurodevelopmental disability (cognitive/motor)

Hypoglycemia and hyperglycemia after birth have proven to be notable determinants that correlate with neurodevelopmental disabilities, both in cognitive and motor domains. A prospective cohort study on 94 term neonates at risk of neonatal encephalopathy concluded a 3.72-fold increased odds of corticospinal tract injury (*P* = .047), 4.82-fold increased odds of the one-point worsened neuromotor score (*P* = .038), and a 15-point lower cognitive and language score on Bayley Scales of Infant Development (*P* = .015) in hypoglycemic neonates.^[60]^ Similarly, in a study on 74 infants, glucose levels were significantly correlated with hearing impairment and neurodevelopmental outcomes at the age of 1: hyperglycemic patients had more hearing impairments than any other group.^[76]^ On the other hand, a single-center retrospective cohort study investigated outcomes in survivors at a longer duration of 2 and 5.5 years. The study reported lower 2-year motor scores and lower motor and cognitive scores for preschool-age infants with hypoglycemia.^[77]^ In another study on 52-term infants, hypoglycemia in the first 6 hours of life was associated with adverse outcomes, defined as death, Griffith Quotient <87, or significant motor disability at 24 months of age.^[48]^ Whilst hyperglycemia has been associated with hearing difficulties, hypoglycemia has been reported to be responsible for most neurodevelopmental disabilities in children with neonatal encephalopathy.

#### 2.3.3. Brain lesions on MRI

Several regions of the brain have been closely examined using MRI to discern the presence of brain lesions among neonates afflicted with encephalopathy. Lesions of white matter, corpus callosum, basal ganglia, cortex, posterior limb internal capsule, and brainstem all were more frequent in glycemic liability and hyperglycemic infants.^[41,76]^ No microstructural changes were associated with hypoglycemia in infants receiving therapeutic hypothermia.^[80]^ In contrast, some previous investigations suggest a strong correlation between hypoglycemia and MRI brain injury, particularly in the posterior gray matter, posterior white matter, and pulvinar nucleus of the thalamus.^[33,78,80]^ However, there is a need for further exploration to conclude with certainty the areas associated with hyperglycemia or hypoglycemia or with both.

#### 2.3.4. Multiorgan dysfunction

In a post hoc analysis of the CoolCap Study, infants with hypoglycemia exhibited more deranged multiorgan parameters when compared to those with hyperglycemia or euglycemia. These hypoglycemic infants had significantly lower pH and bicarbonate levels, higher AST and ALT levels, higher prothrombin time, higher creatinine, and lower urine output during the first 24 hours. In addition, platelet counts were significantly lower, and the heart rate was highest in hypoglycemic neonates. On the other hand, there was no statistically significant difference between partial pressures of oxygen or carbon dioxide, and aEEG parameters.^[13]^ There is, though, a general lack of literature on similar parameters outside of the single study aforementioned.

### 2.4. Glycemic management interventions in neonatal encephalopathy

#### 2.4.1. Therapeutic hypothermia

Therapeutic hypothermia aims to protect the brain by reducing metabolic demands, inflammation, and excitotoxicity.^[81]^ Clinical studies consistently demonstrate improved neurological results in affected infants with hypothermia, reducing the risk of death, disability, cerebral palsy, developmental delay, and cognitive impairment.^[81]^ Initiating therapeutic hypothermia within 6 hours of birth in neonates diagnosed with moderate to severe encephalopathy is crucial for optimizing its effects and improving outcomes.^[81,82]^ While generally well-tolerated, it is essential to be mindful of potential adverse effects such as electrolyte imbalances and cardiovascular instability, necessitating close monitoring and appropriate management to ensure treatment safety and efficacy.^[81,83]^ The neuroprotective benefits of therapeutic hypothermia are well-documented, significantly enhancing long-term neurological prognosis in neonates with encephalopathy.^[81,84]^ A systematic meta-analysis of 11 randomized controlled clinical trials involving 1505 term and near-term infants (≥35-week gestation) with moderate to severe HIE, which evaluated selective head cooling and whole-body cooling initiated within 6 hours of birth, revealed consistently positive effects following hypothermia.^[85]^ In animal studies, delaying the start of cooling significantly diminishes its effectiveness.^[86]^ Research on infant and adult rodents, as well as near-term fetal sheep, has indicated that initiating cooling within 6 hours after hypoxic-ischemic injury (HI) can provide neuroprotection. This protective effect can be achieved by extending the duration of hypothermia to 48 to 72 hours, until secondary phase events like high-amplitude seizures and cytotoxic edema subside.^[87]^ Backing this up, a follow-up cohort study involving 65 surviving newborns who underwent cooling revealed that among them, 43 infants who received cooling before reaching 3 hours of age exhibited notably higher Psychomotor Development Index (PDI) scores, with a median PDI of 90, compared to those cooled after 3 hours, who had a median PDI of 78.^[88]^

Although NE poses the greatest challenge in low- and middle-income countries, there is currently no treatment to reduce the disease burden and fatal outcomes associated with HIE NE. The HELIX study revealed that therapeutic hypothermia did not reduce combined morbidity and mortality in India, Sri Lanka, and Bangladesh, and instead, it significantly raised mortality.^[89]^ Well-executed trials need to be conducted in these regions to test the development of new treatment approaches, facilitated by precise case identifications and subclassification.

#### 2.4.2. Gentamicin therapy

One major cause of neonatal encephalopathy is neonatal sepsis. Gentamicin is commonly administered as an antibiotic to treat suspected or confirmed bacterial infections, including sepsis, pneumonia, and meningitis. These infections can sometimes present with symptoms similar to neonatal encephalopathy, such as lethargy, poor feeding, and abnormal neurological signs, necessitating antibiotic treatment. Gentamicin is not typically used as a primary intervention for neonatal encephalopathy caused by hypoxic-ischemic injury during birth, rather as a precautionary measure to prevent bacterial infections escalating into full-blown neonatal encephalopathy. Therapeutic hypothermia is the standard of care for neonatal encephalopathy, as it has been shown to significantly reduce neurological injury and improve long-term outcomes.^[90]^

#### 2.4.3. Infusion therapy intravenous glucose

In the management of neonates with encephalopathy, glucose infusion plays a supportive role, particularly in cases of hypoglycemia or when nutritional support is required. Glucose infusion should be administered judiciously and with close monitoring of blood glucose levels, especially in neonates with encephalopathy who may have altered metabolic regulation.^[60]^ The decision to use intravenous glucose infusion should be based on clinical indications, ongoing assessment of metabolic status, and consultation with neonatal specialists or pediatricians.^[60]^ Recently, continuous glucose infusion in asymptomatic infants was shown to be therapeutically useful for asymptomatic neonatal hypoglycemia.^[91]^ Administering a glucose infusion (1 gm/kg over 1 hour) effectively reduced the SI by 3.7% (*P* < .001), with no SI rebounding to 7% or more within 24 hours post-infusion.^[92]^

#### 2.4.4. Glucagon therapy

Glucagon, a hormone primarily associated with glucose metabolism, has shown neuroprotective properties in preclinical studies, particularly in hypoxic-ischemic brain injury models. It acts through various mechanisms such as modulation of energy metabolism, anti-inflammatory effects, and promotion of neuronal survival. IV glucagon infusion is recommended by the AUS as it prevents the pancreas from being overly stimulated by rapid glucose infusion and supports uninterrupted breastfeeding.^[93]^ Glucagon has also been found to enhance cerebral blood flow and improve neurological outcomes in animal models of neonatal HIE. In cases of neonatal encephalopathy with metabolic disturbances like hypoglycemia or impaired glucose utilization, glucagon therapy may help maintain sufficient cerebral glucose supply, prevent energy failure, and reduce neuronal damage.

#### 2.4.5. Continuous glucose monitoring

A technology commonly used in diabetes management for real-time glucose level tracking, has shown potential in neonatal encephalopathy. By offering CGM, CGM enables early detection and management of hypoglycemia. Studies have demonstrated the benefits of CGM in neonatal care^[94]^ including improved glycemic control, reduced hypoglycemic episodes^[95]^ and better neurodevelopmental outcomes. CGM allows personalized glucose management, enabling targeted interventions to optimize brain metabolism and reduce neuronal injury in neonatal encephalopathy. Real-time monitoring and timely intervention with CGM have the potential to enhance metabolic stability, prevent complications, and improve long-term neurological outcomes in affected neonates The National Institute for Clinical Excellence now recommends that CGM be offered to adults and children with diabetes who are at risk from hypoglycemia.^[96]^ Several studies have utilized CGM technology for neonates; however, commercially available CGM sensors, such as the Dexcom G6, are FDA-approved and validated only for individuals aged 2 years and older. Despite this, results from multiple studies on neonates are promising, indicating potential for the broader adoption of CGM in neonatal clinical practice.

## 3. Conclusion

Our literature review reported studies that establish a correlation between the severity of dysglycemia and the duration of exposure for adverse outcomes in neonates.^[97]^ The more severe the hyperglycemia, the shorter the exposure duration it takes to produce adverse neurodevelopmental outcomes, such as basal ganglia injury and watershed infarcts. The review finds that over 50% of hypoglycemia and hyperglycemia cases are missed with the current protocols of intermittent glucose monitoring.^[44,97]^ Therefore, the establishment of continuous glucose profile monitoring is essential to ensure dysglycemia is promptly identified in neonates and overtreatment/undertreatment of glycemic interventions is prevented. Our literature review also reports that the ideal glucose range in neonates with encephalopathy is conflicting in different studies.^[98]^ However, the range of dysglycemia with reversible and the most limited adverse effects in neonatal encephalopathy ranges from >2.6 mmol/L to <10.1 mmol/L. Hypoglycemia with glucose levels maintained at >2.6 mmol/L has not been linked with neurological outcomes.^[99]^ Similarly, hyperglycemia maintained at <10.1 mmol/L reduces the incidences of brain injury in neonatal encephalopathy. Despite advancements in understanding the impact of glycemic dysregulation on neonatal encephalopathy, our review is limited in several ways. The exact thresholds of hyperglycemia and hypoglycemia are conflicted in different studies. The conflicting thresholds observed in different studies may stem from variations in study populations, methodologies, or definitions of hyperglycemia and hypoglycemia. The trials we reviewed also reported different methods for glucose monitoring, studied the effects of dysglycemia at various time points, and reported varying outcomes. They were attributed to differences in study designs, patient populations, or the timing of assessments. For example, Kamino et al^[97]^ did not find any association between hypoglycemia and brain injury; however, other studies have established this correlation.^[98,100]^ We did not select studies based on blood samples used for glucose monitoring. Research establishes that the source and type of samples, such as whole blood or blood plasma samples, each affect glucose concentrations.^[101]^ Moreover, the long-term neurodevelopmental outcomes of glycemic interventions, such as therapeutic hypothermia and glucose infusion, remain incompletely understood since most studies report outcomes until 18 months after birth at the most. The majority of the literature studied on brain imaging was observational in nature, so it is hard to prove a definite cause-and-effect relationship between dysglycemia and MRI changes. Further multicenter, prospective research is needed to establish causal relationships and validate the optimal glycemic management interventions. The study of long-term neurodevelopmental outcomes in neonates with hypoglycemia and hyperglycemia is crucial for understanding the full extent of the impact of these glycemic derangements on brain development. Future research should investigate the effects of these glycemic derangements on cognitive, motor, and behavioral outcomes in children beyond the typical 18-month follow-up period. Future research is also needed to investigate the role of glucose variability, insulin resistance, and other metabolic factors in the development of brain injury.

## Author contributions

**Conceptualization:** Sughra Asif.

**Data curation:** Maryam Shaukat.

**Investigation:** Kashaf Khalil.

**Methodology:** Hadiya Javed.

**Project administration:** Muhammad Daim Bin Zafar, Mohammed Mahmmoud Fadelallah Eljack, Muhammad Hasanain.

**Supervision:** Hadiya Javed, Muhammad Daim Bin Zafar, Mohammed Mahmmoud Fadelallah Eljack, Muhammad Hasanain.

**Validation:** Mohammed Mahmmoud Fadelallah Eljack.

**Visualization:** Hadiya Javed.

**Writing – original draft:** Sughra Asif, Maryam Shaukat, Kashaf Khalil, Hadiya Javed, Muhammad Safwan, Khadija Alam, Sabahat Fatima, Prishotam Chohan, Huraim Muhammad Hanif.

**Writing – review & editing:** Mohammed Mahmmoud Fadelallah Eljack, Hadiya Javed, Muhammad Daim Bin Zafar, Muhammad Hasanain.
